# Supplementary material for: Common binding by redundant group B Sox proteins is evolutionarily conserved in Drosophila
Source: BMC Genomics. 2015 Apr 13;16(1):292. doi: 10.1186/s12864-015-1495-3 (PMC4419465; doi:10.1186/s12864-015-1495-3)
Supplement: Additional file 1: Figure S1. — Multiple alignment of Dichaete and SoxNeuro amino acid sequences. (A) Multiple alignment of the entire Dichaete sequence from D. melanogaster, D. simulans, D. yakuba and D. pseudoobscura. (B) Multiple alignment of the entire SoxN sequence from D. melanogaster, D. simulans, D. yakuba and D. pseudoobscura. The HMG domains of each orthologous protein are highlighted in red. [file 12864_2015_1495_MOESM1_ESM.pdf]

A

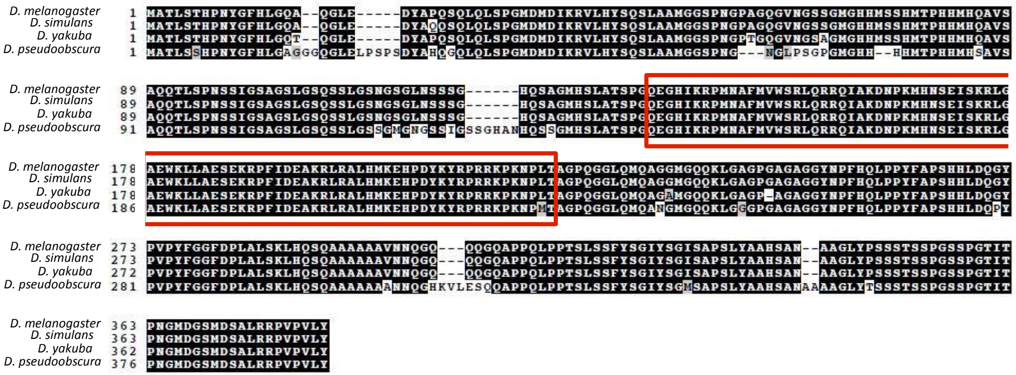
D. melanogaster 1 MATLSTHPNYGFHLGQA--QGLE-----DYAPQSQLQLSPGMDMDIKRVLHYSQSLAAMGGSPNGPAGQGVNGSSGMGHHMSSHMTPHHMHQAVS
D. simulans 1 MATLSTHPNYGFHLGQA--QGLE-----DYAQQSQLQLSPGMDMDIKRVLHYSQSLAAMGGSPNGPAGQGVNGSSGMGHHMSSHMTPHHMHQAVS
D. yakuba 1 MATLSTHPNYGFHLGQT--QGLE-----DYAPQSQLQLSPGMDMDIKRVLHYSQSLAAMGGSPNGPTGQGVNGSAGMGHHMSSHMTPHHMHQAVS
D. pseudoobscura 1 MATLSSHPNYGFHLGAGGGQGLELPSPSDYAHQGQLQLSPGMDMDIKRVLHYSQSLAAMGGSPNG---NGLPSGPGMGHH--HHMTPHHMHSAVS
D. melanogaster 89 AQQTLSPNSSIGSAGSLGSQSSLGSNGSGLNSSSG------HQSAGMHSLATSPGQEGHIKRPMNAFMVWSRLQRRQIAKDNPKMHNSEISKRLG
D. simulans 89 AQQTLSPNSSIGSAGSLGSQSSLGSNGSGLNSSSG------HQSAGMHSLATSPGQEGHIKRPMNAFMVWSRLQRRQIAKDNPKMHNSEISKRLG
D. yakuba 89 AQQTLSPNSSIGSAGSLGSQSSLGSNGSGLNSSSG------HQSAGMHSLATSPGQEGHIKRPMNAFMVWSRLQRRQIAKDNPKMHNSEISKRLG
D. pseudoobscura 91 AQQTLSPNSSIGSAGSLGSQSSLGSSGMGNGSSIGSSGHANHQSSGMHSLATSPGQEGHIKRPMNAFMVWSRLQRRQIAKDNPKMHNSEISKRLG
D. melanogaster 178 AEWKLLAESEKRPFIDEAKRLRALHMKEHPDYKYRPRRKPKNPLTAGPQGGLQMQAGGMGQQKLGAGPGAGAGGYNPFHQLPPYFAPSHHLDQGY
D. simulans 178 AEWKLLAESEKRPFIDEAKRLRALHMKEHPDYKYRPRRKPKNPLTAGPQGGLQMQAGGMGQQKLGAGPGAGAGGYNPFHQLPPYFAPSHHLDQGY
D. yakuba 178 AEWKLLAESEKRPFIDEAKRLRALHMKEHPDYKYRPRRKPKNPLTAGPQGGLQMQAGAMGQQKLGAGP-AGAGGYNPFHQLPPYFAPSHHLDQGY
D. pseudoobscura 186 AEWKLLAESEKRPFIDEAKRLRALHMKEHPDYKYRPRRKPKNPMTAGPQGGLQMQANGMGQQKLGGGPGAGAGGYNPFHQLPPYFAPSHHLDQPY
D. melanogaster 273 PVPYFGGFDPLALSKLHQSQAAAAAAVNNQGQ---QQGQAPPQLPPTSLSSFYSGIYSGISAPSLYAAHSAN--AAGLYPSSSTSSPGSSPGTIT
D. simulans 273 PVPYFGGFDPLALSKLHQSQAAAAAAVNNQGQ---QQGQAPPQLPPTSLSSFYSGIYSGISAPSLYAAHSAN--AAGLYPSSSTSSPGSSPGTIT
D. yakuba 272 PVPYFGGFDPLALSKLHQSQAAAAAAVNNQGQ---QQGQAPPQLPPTSLSSFYSGIYSGISAPSLYAAHSAN--AAGLYPSSSTSSPGSSPGTIT
D. pseudoobscura 281 PVPYFGGFDPLALSKLHQSQAAAAAAANNQGHKVLESQQAPPQLPPTSLSSFYSGIYSGMSAPSLYAAHSANAAAAGLYTSSSTSSPGSSPGTIT
D. melanogaster 363 PNGMDGSMDSALRRPVPVLY
D. simulans 363 PNGMDGSMDSALRRPVPVLY
D. yakuba 362 PNGMDGSMDSALRRPVPVLY
D. pseudoobscura 376 PNGMDGSMDSALRRPVPVLY

B

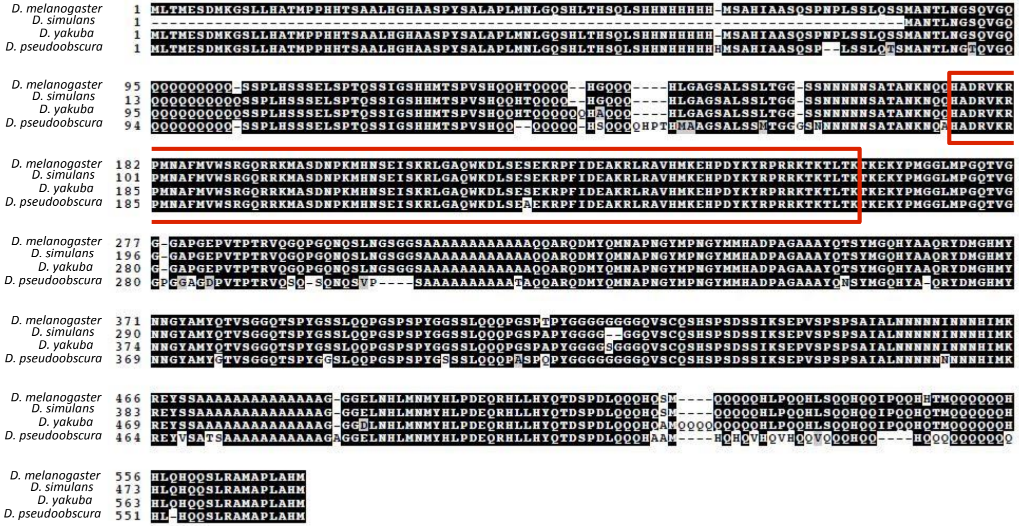
D. melanogaster 1 MLTMESDMKGSLLHATMPPHHTSAALHGHAASPYSALAPLMNLGQSHLTHSQLSHHNHHHHH-MSAHIAASQSPNPLSSLQSSMANTLNGSQVGQ
D. simulans 1 ---MANTLNGSQVGQ
D. yakuba 1 MLTMESDMKGSLLHATMPPHHTSAALHGHAASPYSALAPLMNLGQSHLTHSQLSHHNHHHHH-MSAHIAASQSPNPLSSLQSSMANTLNGSQVGQ
D. pseudoobscura 1 MLTMESDMKGSLLHATMPPHHTSAALHGHAASPYSALAPLMNLGQSHLTHSQLSHHNHHHHHHMSAHIAASQSP--LSSLQTSMANTLNGTQVGQ
D. melanogaster 95 QQQQQQQQQ-SSPLHSSSELSPTQSSIGSHHMTSPVSHQQHTQQQQ--HGQQQ----HLGAGSALSSLTGG-SSNNNNNSATANKNQQHADRVKR
D. simulans 13 QQQQQQQQQQSSPLHSSSELSPTQSSIGSHHMTSPVSHQQHTQQQQ--HGQQQ----HLGAGSALSSLTGG-SSNNNNNSATANKNQQHADRVKR
D. yakuba 95 QQQQQQQQQQSSPLHSSSELSPTQSSIGSHHMTSPVSHQQHTQQQQQHAQQQ----HLGAGSALSSLTGG-SSNNNNNSATANKNQQHADRVKR
D. pseudoobscura 94 QQQQQQQQQ-SSPLHSSSELSPTQSSIGSHHMTSPVSHQQ--QQQQQ-HSQQQQHPTHMAAGSALSSMTGGGSNNNNNNSATANKNQAHADRVKR
D. melanogaster 182 PMNAFMVWSRGQRRKMASDNPKMHNSEISKRLGAQWKDLSESEKRPFIDEAKRLRAVHMKEHPDYKYRPRRKTKTLTKTKEKYPMGGLMPGQTVG
D. simulans 101 PMNAFMVWSRGQRRKMASDNPKMHNSEISKRLGAQWKDLSESEKRPFIDEAKRLRAVHMKEHPDYKYRPRRKTKTLTKTKEKYPMGGLMPGQTVG
D. yakuba 185 PMNAFMVWSRGQRRKMASDNPKMHNSEISKRLGAQWKDLSESEKRPFIDEAKRLRAVHMKEHPDYKYRPRRKTKTLTKTKEKYPMGGLMPGQTVG
D. pseudoobscura 185 PMNAFMVWSRGQRRKMASDNPKMHNSEISKRLGAQWKDLSEAEKRPFIDEAKRLRAVHMKEHPDYKYRPRRKTKTLTKTKEKYPMGGLMPGQTVG
D. melanogaster 277 G-GAPGEPVTPTRVQGQPGQNQSLNGSGGSAAAAAAAAAAAAAQQARQDMYQMNAPNGYMPNGYMMHADPAGAAAYQTSYMGQHYAAQRYDMGHMY
D. simulans 196 G-GAPGEPVTPTRVQGQPGQNQSLNGSGGSAAAAAAAAAAAAAQQARQDMYQMNAPNGYMPNGYMMHADPAGAAAYQTSYMGQHYAAQRYDMGHMY
D. yakuba 280 G-GAPGEPVTPTRVQGQPGQNQSLNGSGGSAAAAAAAAAAAAAQQARQDMYQMNAPNGYMPNGYMMHADPAGAAAYQTSYMGQHYAAQRYDMGHMY
D. pseudoobscura 280 GPGGAGDPVTPTRVQSQ-SQNQSVP----SAAAAAAAAAATAQQARQDMYQMNAPNGYMPNGYMMHADPAGAAAYQNSYMGQHYA-QRYDMGHMY
D. melanogaster 371 NNGYAMYQTVSGGQTSPYGSSLQQPGSPSPYGGSSLQQQPGSPTPYGGGGGGGGQVSCQSHSPSDSSIKSEPVSPSPSAIALNNNNNINNNHIMK
D. simulans 290 NNGYAMYQTVSGGQTSPYGSSLQQPGSPSPYGGSSLQQQPGSPAPYGGGG--GGQVSCQSHSPSDSSIKSEPVSPSPSAIALNNNNNINNNHIMK
D. yakuba 374 NNGYAMYQTVSGGQTSPYGSSLQQPGSPSPYGGSSLQQQPGSPAPYGGGGSGGGQVSCQSHSPSDSSIKSEPVSPSPSAIALNNNNNINNNHIMK
D. pseudoobscura 369 NNGYAMYGTVSGGQTSPYGGSLQQPGSPSPYGSSSLQQQPASPQPYGGGGGGGGQVSCQSHSPSDSSIKSEPVSPSPSAIALNNNNNNNNNHIMK
D. melanogaster 466 REYSSAAAAAAAAAAAAAAG-GGELNHLMNMYHLPDEQRHLLHYQTDSPDLQQQHQSM----QQQQQHLPQQHLSQQHQQIPQQHHTMQQQQQQH
D. simulans 383 REYSSAAAAAAAAAAAAAAG-GGELNHLMNMYHLPDEQRHLLHYQTDSPDLQQQHQSM----QQQQQHLPQQHLSQQHQQIPQQHQTMQQQQQQH
D. yakuba 469 REYSSAAAAAAAAAAAAAAG-GGDLNHLMNMYHLPDEQRHLLHYQTDSPDLQQQHQAMQQQQQQQQQHLPQQHLSQQHQQIPQQHQTMQQQQQQH
D. pseudoobscura 464 REYVSATSAAAAAAAAAAAGAGGELNHLMNMYHLPDEQRHLLHYQTDSPDLQQQHAAM----HQHQVHQVHQQVQQQHQQ----HQQQQQQQQQ
D. melanogaster 556 HLQHQQSLRAMAPLAHM
D. simulans 473 HLQHQQSLRAMAPLAHM
D. yakuba 563 HLQHQQSLRAMAPLAHM
D. pseudoobscura 551 HL-HQQSLRAMAPLAHM
