# Supplementary figures and images for: Common binding by redundant group B Sox proteins is evolutionarily conserved in Drosophila
Source: BMC Genomics. 2015 Apr 13;16(1):292. doi: 10.1186/s12864-015-1495-3 (PMC4419465; doi:10.1186/s12864-015-1495-3)

**A**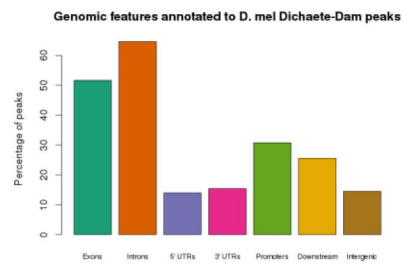**C**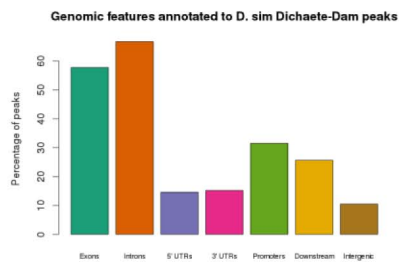**E**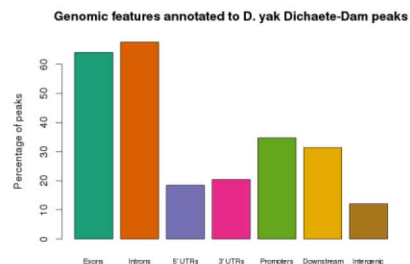**B**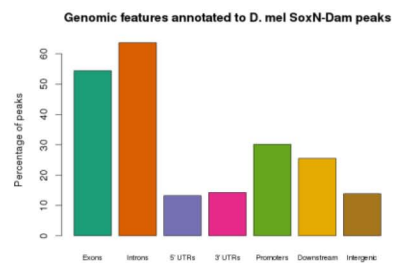**D**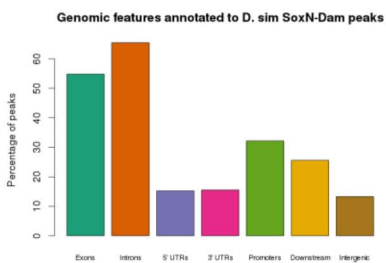**F**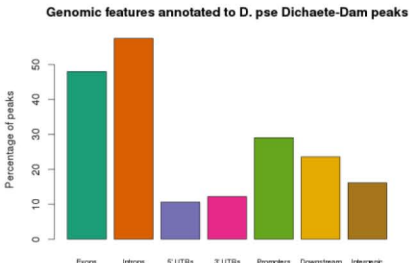**G**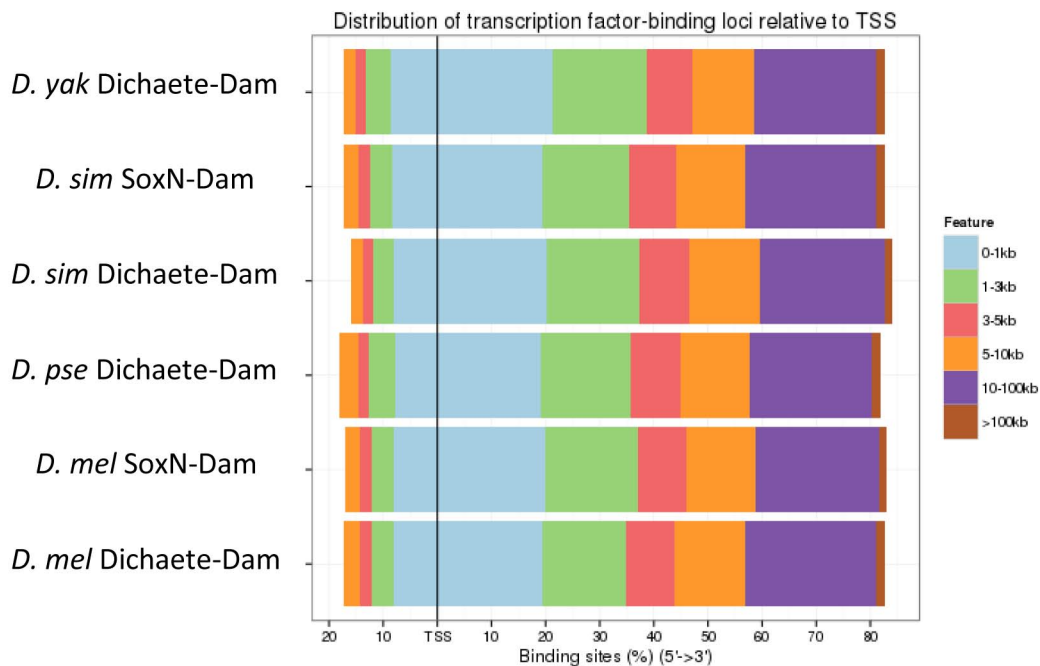

Supplement: Additional file 4: Figure S2. — Genomic features annotated to group B Sox DamID binding intervals. Feature classes include exons, introns, 5′ UTRs, 3′UTRs, promoters, immediate downstream and intergenic. Each interval may be annotated with more than one class if it overlaps multiple features. (A) Percentages of D. melanogaster Dichaete-Dam intervals annotated to each feature class. (B) Percentages of D. melanogaster SoxN-Dam intervals annotated to each feature class. (C) Percentages of D. simulans Dichaete-Dam intervals annotated to each feature class. (D) Percentages of D. simulans SoxN-Dam intervals annotated to each feature class. (E) Percentages of D. yakuba Dichaete-Dam intervals annotated to each feature class. (F) Percentages of D. pseudoobscura Dichaete-Dam intervals annotated to each feature class. (G) Plot of the distribution of distances to the nearest TSS for each binding dataset according to the scale on the right. [file 12864_2015_1495_MOESM4_ESM.pdf]

**A**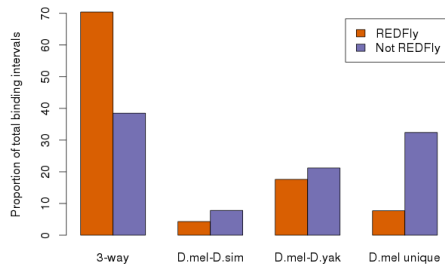**C**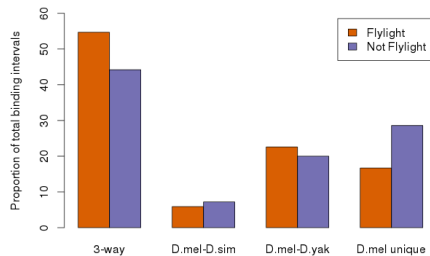**B**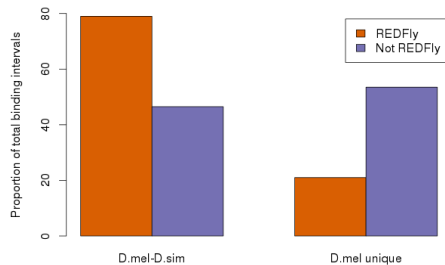**D**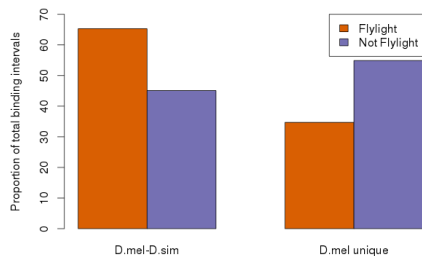

Supplement: Additional file 7: Figure S3. — DamID intervals overlapping a known CRM are preferentially conserved. (A) Dichaete-Dam binding intervals that overlap a REDFly CRM are more likely to show three-way binding conservation between D. melanogaster, D. simulans and D. yakuba (“3-way”) and are less likely to be unique to D. melanogaster (“D. mel unique”) than those that do not (p = 7.06e-35). (B) SoxN-Dam binding intervals that overlap a REDFly CRM are more likely to show two-way binding conservation between D. melanogaster and D. simulans (“D.mel-D.sim”) and are less likely to be unique to D. melanogaster than those that do not (p = 2.40e-72). (C) Dichaete-Dam binding intervals that overlap a FlyLight CRM are more likely to show three-way binding conservation and are less likely to be unique to D. melanogaster than those that do not (p = 3.38e-38). (D) SoxN-Dam binding intervals that overlap a FlyLight CRM are more likely to show two-way binding conservation and are less likely to be unique to D. melanogaster than those that do not (p = 7.27e-12). [file 12864_2015_1495_MOESM7_ESM.pdf]

**A**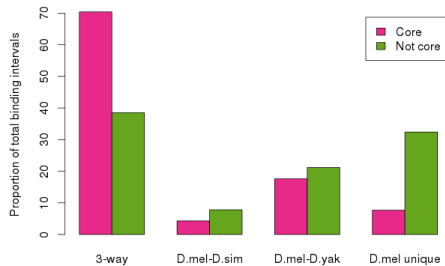**C**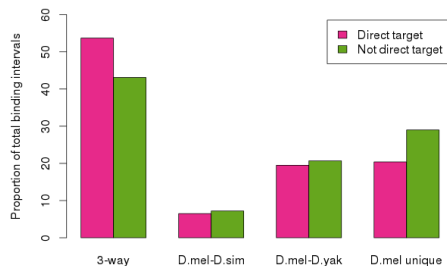**B**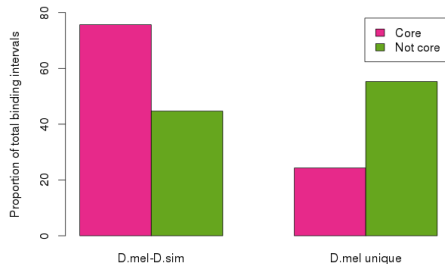**D**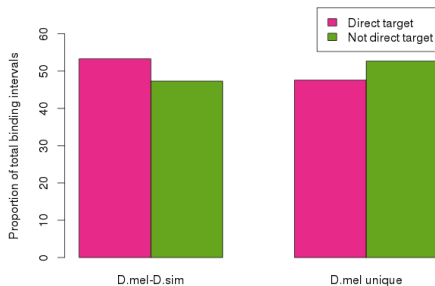

Supplement: Additional file 8: Figure S4. — DamID intervals overlapping a core binding interval or annotated to a direct target gene are preferentially conserved. (A) Dichate-Dam binding intervals that overlap a core Dichaete binding site are more likely to show three-way conservation between D. melanogaster, D. simulans and D. yakuba (“3-way”) and are less likely to be unique to D. melanogaster (“D. mel unique”) than those that do not (p = 4.10e-305). (B) SoxN-Dam binding intervals that overlap a core SoxN binding site are more likely to show two-way conservation between D. melanogaster and D. simulans (“2-way”) and are less likely to be unique to D. melanogaster than those that do not (p = 1.90e-161). (C) Dichaete-Dam binding intervals that are annotated to a Dichaete direct target gene are more likely to show three-way conservation and are less likely to be unique to D. melanogaster than those that are not (p = 2.3e-12). However, this effect is weaker than for core intervals. (D) SoxN-Dam binding intervals that are annotated to a SoxN direct target gene are more likely to show two-way conservation and are less likely to be unique to D. melanogaster than those that are not (p = 3.4e-5). Again, however, this effect is weaker than for core intervals. [file 12864_2015_1495_MOESM8_ESM.pdf]
